# When an earthquake hits a war-affected population: longitudinal growth trajectories of psychopathology in individuals in northwest Syria

**DOI:** 10.1192/bjp.2025.1

**Published:** 2026-03

**Authors:** Dana Churbaji, Linnea Ritter, Pascal Schlechter, Ahlke Kip, Nexhmedin Morina

**Affiliations:** Institute of Psychology, University of Münster, Germany; Department of Psychology, New School for Social Research, USA

**Keywords:** Anxiety or fear-related disorders, comorbidity, depressive disorders, longitudinal data, trauma and stressor-related disorders

## Abstract

**Background:**

The Kahramanmaraş earthquakes in February 2023 represented a disaster within a disaster, as northwest Syria had been affected by years of war. Literature on the immediate psychological impact of such natural disasters in high-adversity populations is lacking.

**Aims:**

To examine prevalences, longitudinal trajectories and cognitive predictors of post-traumatic stress disorder (PTSD), depression and generalised anxiety disorder (GAD) in survivors of armed conflict in northwest Syria exposed to the Kahramanmaraş earthquakes.

**Method:**

We assessed self-reported PTSD, depression and GAD symptoms, as well as self-efficacy and repetitive negative thinking (RNT), at 4, 11 and 18 weeks post-earthquake (T1, T2 and T3, respectively) in 204 war survivors exposed to recent earthquakes. Retention rates for T2 and T3 were 84.4 and 75.8%, respectively. To determine trajectories of PTSD, depression and GAD, we conducted latent class growth analyses with time, self-efficacy and RNT as predictors, and trauma history, education and gender as covariates.

**Results:**

Prevalences of probable PTSD, depression and GAD according to questionnaire cut-offs were 80.4, 79.9 and 70.1% at T1; 62.2, 57.2 and 54.2% at T2; and 62.1, 55.2 and 51.1% at T3. Across all disorders, three developmental trajectories emerged, with most participants following a recovery or low-symptom trajectory. RNT was associated with protracted recovery.

**Conclusions:**

Natural disasters are associated with poor mental health in individuals in war-torn regions. Although latent class growth analyses indicated prevailing recovery trajectories, prevalence remained alarmingly high across time. RNT emerged as a potential transdiagnostic factor across disorders. Research and interventions should prioritise northwest Syrians’ unprecedented mental health needs.

In February 2023, the Kahramanmaraş earthquakes struck northwest Syria while temperatures were well below zero. The earthquakes represented a disaster within a disaster, as years of war, violence, poor living conditions, displacement and limited access to healthcare had created sociopolitical challenges that hindered rescue efforts.^1^

## Mental health consequences of armed conflict and natural disasters

The convergence of armed conflict and natural disasters has been found to be associated with long-term mental health consequences in both longitudinal and cross-sectional research, although prevalence estimates vary widely. Cross-sectional studies in low- and middle-income countries (LMICs) affected by armed conflicts and natural disasters, such as Vietnam^2,3^ and Pakistan,^4^ have indicated that 6.9^2^ to 64.6%^4^ of affected people have post-traumatic stress disorder (PTSD), 6^3^ to 12.7%^2^ have depression, and 2% have generalised anxiety disorder (GAD).^3^ Further studies in various settings and samples, such as children and veterans, have reported similarly high yet heterogenous prevalences following exposure to conflicts and natural disasters.^5–7^ Some studies have also determined longitudinal mental health trajectories in these populations. A 12-month longitudinal study in Sri Lanka, conducted 10 years after the tsunami and 6 years post-conflict, assessing anxiety and depression, reported a recovery trajectory in 23.5% of the sample, a persistently low-symptom trajectory in 19.7%, and symptomatic trajectories in more than half (56.8%). This study also identified higher trauma exposure and female gender as significant risk factors for poor mental health.^8^ In East Timor, a region grappling with recurrent conflict and adversities, a 6-year longitudinal study identified four trajectories of PTSD: low symptom (34.7%), recovery (20.8%), persistent symptom (7.2%) and new onset (37.2%). Family conflict and higher trauma exposure were found to be risk factors for the persistent-symptom and new-onset trajectories.^9^ Despite high levels of psychopathology observed in affected regions, a substantial percentage of survivors exhibited psychological resilience.^8,9^ Yet, there is a lack of longitudinal studies on the immediate aftermath of natural disasters in settings affected by large-scale conflict. Such studies could increase our understanding of the temporal course of psychopathology and potentially related cognitive mechanisms in affected survivors.

## The impact of the Kahramanmaraş earthquakes

Regarding the Kahramanmaraş earthquakes, two cross-sectional studies in Turkey assessed PTSD in out-patient^10^ and student^11^ samples 12 and 16 weeks post-earthquakes and reported prevalence of 51.4 and 43.5%, respectively. Further, a study conducted 40 days after the earthquakes in a regime-held part of Syria reported prevalences of 45% for PTSD, 51% for depression and 48% for GAD.^12^ However, specific factors affecting the population in northwest Syria include the ongoing armed conflict, control by fragmented and complex non-state actors, and a high proportion of internally displaced people with a history of trauma exposure and elevated levels of psychopathology.^1^ To date, no data are available for this high-adversity population.

## Longitudinal trajectories and underlying cognitive predictors

Beyond prevalence estimates, it is essential to examine symptom trajectories over time. Identifying individuals with similar trajectories will provide insight into between-person differences in spontaneous remission or chronic development, enabling tailored interventions to address specific needs.^13^ Investigating cognitive predictors of these trajectories is crucial in this context.^14,15^ Self-efficacy, an individual’s belief in their capability to cope with challenges and achieve desired outcomes, can have a significant role in post-disaster recovery.^14^ High self-efficacy can promote adaptive coping strategies and buffer psychopathology.^16^ Repetitive negative thinking (RNT) manifests as a maladaptive repetitive focus on negative content and is perceived as difficult to control. It has been identified as a transdiagnostic cognitive process relevant to the development and maintenance of psychopathology.^15,17^ Examining self-efficacy and RNT in relation to PTSD, depression and GAD symptom trajectories can provide a more holistic perspective on the mental health impact of earthquakes in a war-affected population.

## Study aims

The present study aimed to examine prevalences and longitudinal trajectories of PTSD, depression and GAD, as well as underlying cognitive mechanisms, in war survivors in northwest Syria shortly after the earthquakes.^18^ First, we examined prevalences of PTSD, depression and GAD across three waves among adult earthquake survivors in Jisr-Alshughur (Idlib, northwest Syria). Considering our aim to examine mental health consequences of a natural disaster in the context of ongoing mass violence, we expected relatively high prevalences of PTSD, depression and GAD that would remain stable over the course of 18 weeks post-earthquake. Our second objective was to identify distinct trajectories of these conditions. Including RNT and self-efficacy as predictors enabled us to assess the impact of time while controlling for both levels of and changes in self-efficacy and RNT. Drawing on previous longitudinal studies,^8^ we expected to identify three symptom trajectories – high distress, recovery and low symptom – with higher levels of RNT predicting greater symptom severity and high levels of self-efficacy predicting lower symptom levels across all trajectories. Gender, education and trauma history were included as covariates to enable us to better understand the profiles of individuals within each trajectory class. Specifically, we expected the high-distress trajectory to be associated with higher levels of trauma history, female gender and lower education levels compared with the recovery and low-symptom trajectories.

## Method

### Study design

We conducted a three-wave longitudinal study to examine associations of self-efficacy and RNT with trajectories of PTSD, depression and GAD. Ethical approval was received from the local ethics committee of the University of Münster, Germany (reference 2023-20-DCh). We assert that all procedures contributing to this work comply with the ethical standards of the relevant national and institutional committees on human experimentation and with the Helsinki Declaration of 1975, as revised in 2013. We followed the STROBE guidelines for observational studies.^19^ Participants were contacted in Jisr-Alshughur, Syria, a region exposed to long-lasting armed conflicts and recent earthquakes.^1^ Recruitment difficulties were amplified in the region owing to its complex political climate, which is marked by restrictive authorities, insufficient infrastructure and a need to establish community trust. Aiming to complete the first wave in the immediate aftermath of the earthquakes (within a 4-week timeframe) further limited the time available for preparation and recruitment. We therefore applied non-probability snowball sampling and did not perform *a priori* power analysis. A local team of two men and one woman initiated recruitment by engaging community members and encouraging referrals through their social networks. We sought to ensure a sample size for stable parameter estimates and aimed for approximately 200 participants at each wave.^20^ This study is part of an ongoing project that includes an online survey comprising ten questionnaires, with an anticipated duration of 18 minutes. Skipping questions or navigating back in the survey is not possible. Written informed consent was obtained from all participants at the beginning of the survey. Inclusion criteria were: (a) residing in northwest Syria and (b) having experienced the Kahramanmaraş earthquakes. Exclusion criteria were (a) being younger than 18 years old, (b) reporting a diagnosis of a psychotic disorder, (c) reporting current suicide risk and (d) lacking informed consent. If participants withheld consent or did not meet inclusion criteria, the survey would automatically conclude with contact information for the mental health hotline of the Syrian Association for Mental Health. Participants received compensation after each assessment, totalling $10.

### Participants

The initial data collection (time point 1; T1) took place 4 weeks after the main shocks, with most participants (77.5%) living in tents under challenging conditions. The two subsequent waves of data collections were conducted at 7-week intervals, specifically at 11 and 18 weeks post-earthquakes (T2 and T3, respectively). Completion rates were 74.0% at T1, 75.8% at T2 and 82.9% at T3. Of the initial *N* = 334 participants who completed the survey at T1, *n* = 282 (84.4%) participated at T2, and *n* = 253 (75.8%) participated at T3. Little’s missing completely at random test indicated no systematic data missingness for our variables of interest (PTSD, depression, GAD, trauma history, self-efficacy, RNT, gender and education status), χ^2^(33)=37.59, *P* = 0.27. Further, chi-squared tests and one-way analyses of variance revealed no significant association of gender, education or age with dropout at T2 or T3. To ensure data quality, participants who completed the survey in less than 8 min at T1 or less than 7 min at T2 and T3, were excluded, as this led us to suspect a lack of attention.^21^ This exclusion left 263, 220 and 192 participants for T1, T2 and T3, respectively. Participants who changed their identified gender across waves were further excluded, yielding *n* = 243, *n* = 201 and *n* = 174 participants for T1, T2 and T3, respectively. Missing values for participants completing two of the three waves were accounted for using the full information likelihood method provided by Latent Gold 6.0.^22^ However, *n* = 39 participants only completing T1 were excluded, as their missing data for the later waves might not have been fully explainable by observed variables from T1. Therefore, the final sample sizes were *N* = 204 for T1, *N* = 201 for T2 and *N* = 174 for T3. Participants were on average 34.0 years old (s.d. 10.3), 57.4% were female, 60.8% had ≤12 years of education and 95.6% reported experiencing war at close quarters (Table 1).

**Table 1:** Sample characteristics

	T1 (*n* = 204)	T2 (*n* = 201)	T3 (*n* = 174)
Sociodemographic characteristics			
Gender			
Female (%)	57.4	57.7	56.9
Age, years			
Mean (s.d.)	34.05 (10.3)	34.17 (10.32)	34.41 (10.5)
Education			
≤6 years (%)	13.2	12.9	14.4
7–9 years (%)	28.4	28.9	26.3
10–12 years (%)	19.1	18.9	19.5
>12 years (%)	39.2	39.3	39.7
Mean (s.d.)	10.52 (2.71)	10.52 (2.71)	10.50 (2.78)
Living situation			
Own house (%)	15.2	31.8	42.5
Own tent (%)	59.8	51.7	38.5
House of relatives (%)	6.9	8.5	14.4
Tent of relatives (%)	18.1	8.0	4.6
Trauma history			
War at close quarters (%)	95.6	95.5	94.8
Extreme cold weather with no shelter (%)	65.2	64.7	64.4
Lack of food (hunger or fear of hunger) (%)	63.7	63.2	63.8
Lack of healthcare (%)	63.2	62.7	62.6
Loss of family member(s) or loved one(s) (%)	61.8	62.2	61.5
Forced separation from family or close friends (%)	44.6	44.8	46.6
Kidnapping or arrest (%)	33.8	33.8	33.3
Severe accident (car accident or similar) (%)	32.8	32.3	32.8
Witnessing physical violence or assault (%)	31.9	31.3	31.6
Life-threatening illness or injury (%)	27.0	26.9	25.3
Torture (%)	15.7	14.9	14.9
Physical violence or assault (%)	8.8	8.5	8.0
Sexual violence (%)	2.5	2.5	2.9
Mean (s.d.)	6.41 (3.05)	6.37 (3.05)	6.36 (3.02)
Mental disorders			
ITQ			
Prevalence (%)	80.4	62.2	62.1
Mean (s.d.)	14.57 (4.48)	11.66 (4.78)	11.53 (4.78)
PHQ-8			
Prevalence (%)	79.9	57.2	55.2
Mean (s.d.)	14.01 (5.21)	10.66 (5.47)	10.42 (5.34)
GAD-7			
Prevalence (%)	70.1	54.2	51.1
Mean (s.d.)	12.63 (5.0)	10.32 (5.19)	9.51 (4.97)
Cognitive variables			
GSE			
Mean (s.d.)	26.88 (5.22)	26.58 (5.42)	27.11 (4.79)
PTQ			
Mean (s.d.)	50.55 (10.89)	46.88 (12.41)	46.17 (13.12)

GAD-7, Generalised Anxiety Disorder Scale-7; GSE, General Self-Efficacy scale; ITQ, International Trauma Questionnaire; PHQ-8, Patient Health Questionnaire-8; PTQ, Perseverative Thinking Questionnaire. T1 (time point 1), 4 weeks post-earthquake; T2, 11 weeks post-earthquake; T3: 18 weeks post-earthquake.

### Variables

#### Demographic variables

Age, education and potentially traumatic experiences were assessed at T1. Potentially traumatic experiences were assessed using Sigvardsdotter’s^23^ Refugee Trauma History Checklist, with additional items as listed in Table 1. Across all three waves, we assessed gender to ensure data quality and living conditions (tent versus house).

#### Choice of primary measures

To assess PTSD, we used the Arabic version of the International Trauma Questionnaire,^24^ which is based on the ICD-11 and consists of six items (range 0–4). In the present study, the International Trauma Questionnaire was used to capture re-experiencing, avoidance and current threat perceptions tied to the earthquakes (e.g. ‘Having upsetting dreams related to the earthquakes?’). A cut-off score of ≥2 on at least one symptom from each of the three clusters indicates potential PTSD. We assessed depression using the nine-item Patient Health Questionnaire (PHQ-9), applying the suicidality item to meet the inclusion criterion. For analysis, we used the PHQ-8, which excludes the suicidality item but maintains comparable psychometric properties.^25^ GAD was assessed using the seven-item Generalised Anxiety Disorder Scale (GAD-7).^26^ Both the PHQ-8 and the GAD-7 have been validated in Arabic by Sawaya et al.^27^ Items are rated on a four-point Likert scale (range 0–3), with higher cumulative scores indicating greater symptom severity. A cut-off score of ≥10 is recommended for probable diagnosis and has been validated across diverse cultural contexts.^28^

Regarding predictors, the ten-item General Self-Efficacy Scale^29^ measures individuals’ confidence in overcoming obstacles and adapting to difficult or novel situations, using a four-point Likert scale (range 1–4). Reliability and validity of the Arabic version have been established.^30^ RNT was assessed with the 15-item Perseverative Thinking Questionnaire.^31,32^ This disorder-independent measure employs a five-point Likert scale (range 0–4) to measure five facets of RNT: repetitiveness, intrusiveness, difficulty disengaging, unproductiveness and mental capacity capture. Internal consistencies across the three waves were good (all α ≥ 0.81) for all questionnaires (Supplementary Table 1 available at https://doi.org/10.1192/bjp.2025.1).

### Analysis

As preregistered (https://osf.io/6ymjr), we initially planned a cross-lagged panel analysis (CLPA) across three data waves to assess bidirectional associations among the assessed variables. However, owing to poor model fit of the initial models, we shifted to latent class growth analysis (LCGA), a growth mixture modelling approach.^33^ Unlike CLPA, which assumes the same growth pattern, LCGA allows for varied growth trajectories among different groups, while considering covariates (i.e. trauma history, gender and education). LCGA’s flexibility and fewer assumptions help to uncover unique patterns that might be overlooked with CLPA. LCGA identifies groups (latent classes) that share similar growth trajectories over time, highlighting longitudinal between-person differences. Although LCGA accounts for within-person changes, these are conceptualised as uniform within each latent class, and interclass differences are emphasised. The distribution of individual temporal trajectories accounts for between-subject heterogeneity and enables examination of both within- and between-subject variance in the outcome. Analyses were conducted using SPSS version 28.0 (IBM) and Latent Gold 6.0.^22^ Dependent variables (PTSD, depression and GAD) were treated as continuous. Predictors were time (nominal), self-efficacy (numeric) and RNT (numeric). Covariates were gender (nominal), trauma history (numeric) and education (numeric). Conceptually, predictors explain differences in trajectory development, whereas covariates adjust for factors influencing class membership probabilities. To determine the optimal number of latent classes, a single-class model was fitted and then sequentially tested against models with increasing latent classes. Model fit was evaluated based on the log-likelihood, Akaike information criterion (AIC), AIC with three penalising factors (AIC3) to correct for small sample size and prevent overfitting, and Bayesian information criterion (BIC), with lower values indicating better fit. The Vuong–Lo–Mendell–Rubin likelihood ratio test (VLMR) was also used; a significant VLMR (*P* < 0.05) suggest that a model with *k* classes outperforms the previous one with *k* − 1 classes. Explained variance (*R* ^2^), entropy (classification quality), theoretical interpretability and parsimony were also considered as model selection criteria.

## Results

Trauma history was consistently associated with GAD across all waves but correlated with PTSD only at T1. PTSD, depression and GAD were significantly positively intercorrelated over the three waves (Supplementary Table 2).

### Prevalences

As shown in Table 1, at T1 we consistently found elevated prevalences of all disorders (80.4% for PTSD, 79.9% for depression and 70.1% for GAD). There was a noticeable decline at T2 (62.2% for PTSD, 57.2% for depression and 54.2% for GAD) that remained relatively stable into T3 (62.1% for PTSD, 55.2% for depression and 51.1% for GAD). Overall, more than half of the sample scored above the cut-off score for diagnosis of these conditions 18 weeks post-earthquakes. At T1, 81.9% of participants met criteria for at least two disorders, decreasing to 57.2% at T2 and 55.2% at T3.

### Latent class growth analysis

In all three LCGAs (PTSD, depression and GAD), a three-class model showed the best fit. For PTSD, compared with the two-class model, the three-class model had (a) lower AIC and AIC3 values, (b) significant VLMR value, (c) lower classification error value and (d) greater explained variance (*R* ^2^ = 0.68). Subsequent models showed little improvement, and there was a non-significant VLMR result for the four-class model. Analyses for depression mirrored this pattern, with increasing AIC3 values and a non-significant VLMR in the four-class model. In the GAD analysis, although AIC and AIC3 reductions were consistent from the two-class to the three-class model and from the three-class to the four-class model, the decreased entropy and diminishing increase in *R* ^2^ in the four-class model favoured the three-class model (Supplementary Table 3).

**Fig. 1:**
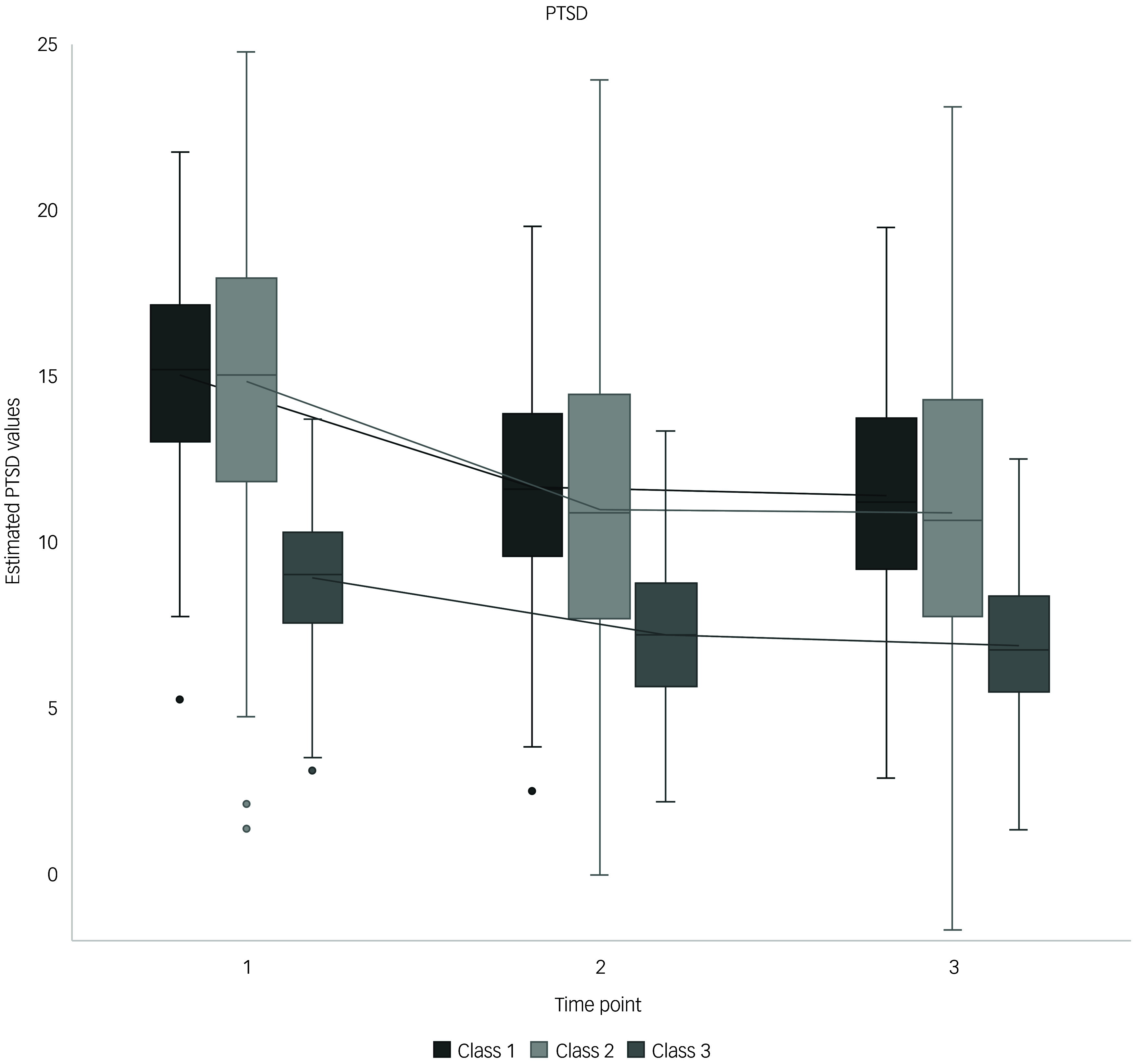
Estimated post-traumatic stress disorder (PTSD) trajectories against time, controlled for self-efficacy and repetitive negative thinking. Class 1, recovery trajectory; class 2, low symptom trajectory; class 3, high distress trajectory. Time point 1, 4 weeks post-earthquake; time point 2, 11 weeks post-earthquake; time point 3: 18 weeks post-earthquake.

The three PTSD trajectories explained 67.8% of the total variance: recovery (class 1), low symptom (class 2) and high distress (class 3), with class distributions of 59.9, 35.6 and 4.5%, respectively (Supplementary Table 4). Among predictors, time significantly differentiated only the class 3 trajectory, whereas RNT differentiated all trajectories. Self-efficacy had no significant impact. Trauma history emerged as a significant covariate, whereas gender and education did not significantly influence class membership. Class 1 had a moderate intercept of 5.59, indicating the predicted PTSD symptom level when all predictors were set to zero. Participants with this profile were characterised by a greater trauma history (0.41). Class 2 had the lowest intercept (0.95) and similar symptom decline, with participants reporting a lesser trauma history (0.09). In both classes, symptoms decreased from T2 to T3, and higher RNT values were associated with increased symptoms. Class 3 had the highest intercept (16.77) and showed a symptom increase at T2, followed by a decrease at T3. Time significantly differentiated symptom development in this trajectory, whereas higher RNT rates predicted lesser symptoms, and trauma history reduced the likelihood of belonging to this class (−0.50). Despite the small size of class 3, its parameters remained similar in a four-class model, signifying its stability. Figure 1 shows estimated PTSD trajectories over time, adjusted for changes in both self-efficacy and RNT.

**Fig. 2:**
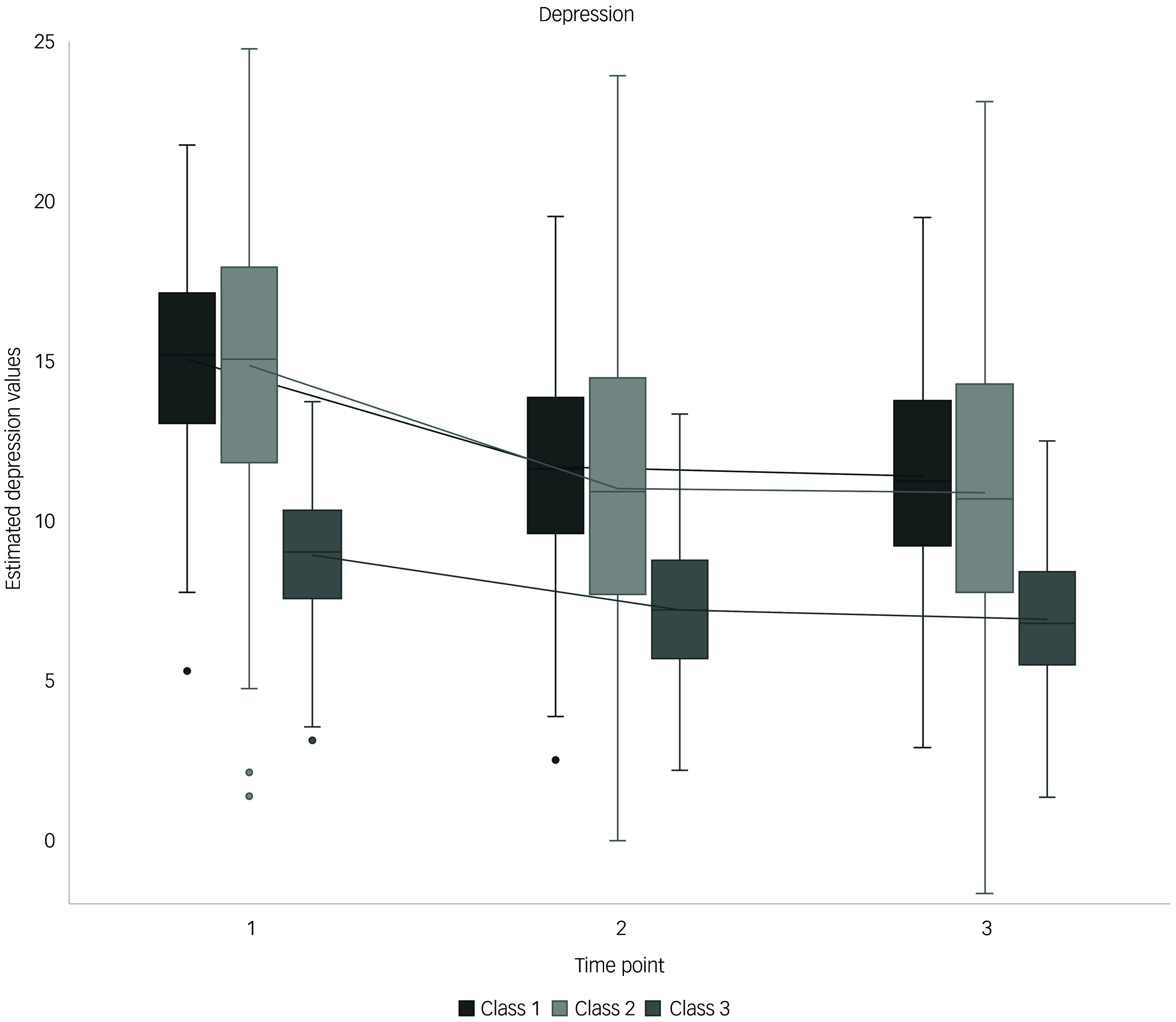
Estimated depression trajectories against time, controlled for self-efficacy and repetitive negative thinking (RNT). Class 1, high trauma history – recovery; class 2, RNT – sensitive recovery; class 3, high distress. Time point 1, 4 weeks post-earthquake; time point 2, 11 weeks post-earthquake; time point 3: 18 weeks post-earthquake.

For depression, the three trajectories identified explained 64.9% of the total variance. Starting from T2, time exhibited negative coefficients for all classes, indicating general recovery, with no significant differences in development over time; this led to the following classification: ‘high trauma history – recovery’ (class 1), ‘RNT - sensitive recovery’ (class 2) and ‘high distress’ (class 3), with class distributions of 63.3, 20.6 and 16.2%, respectively (Supplementary Table 5). The intercept did not significantly differentiate between classes, with class 3 showing the highest intercept value (3.9). Whereas self-efficacy showed no significant impact, RNT emerged as a significant predictor; it most strongly influenced class 2, in which it increased symptoms by 0.4, followed by class 1 with an increase of 0.27 and class 3 with an increase of 0.18. Greater trauma history increased the likelihood of being in class 1 (0.85) or class 3 (0.09) but decreased that of being in class 2 (−0.95). Participants in class 2 had significantly lower education levels than those in classes 1 and 3. Gender did not influence class membership. Figure 2 illustrates the depression trajectories over time, adjusted for self-efficacy and RNT.

**Fig. 3:**
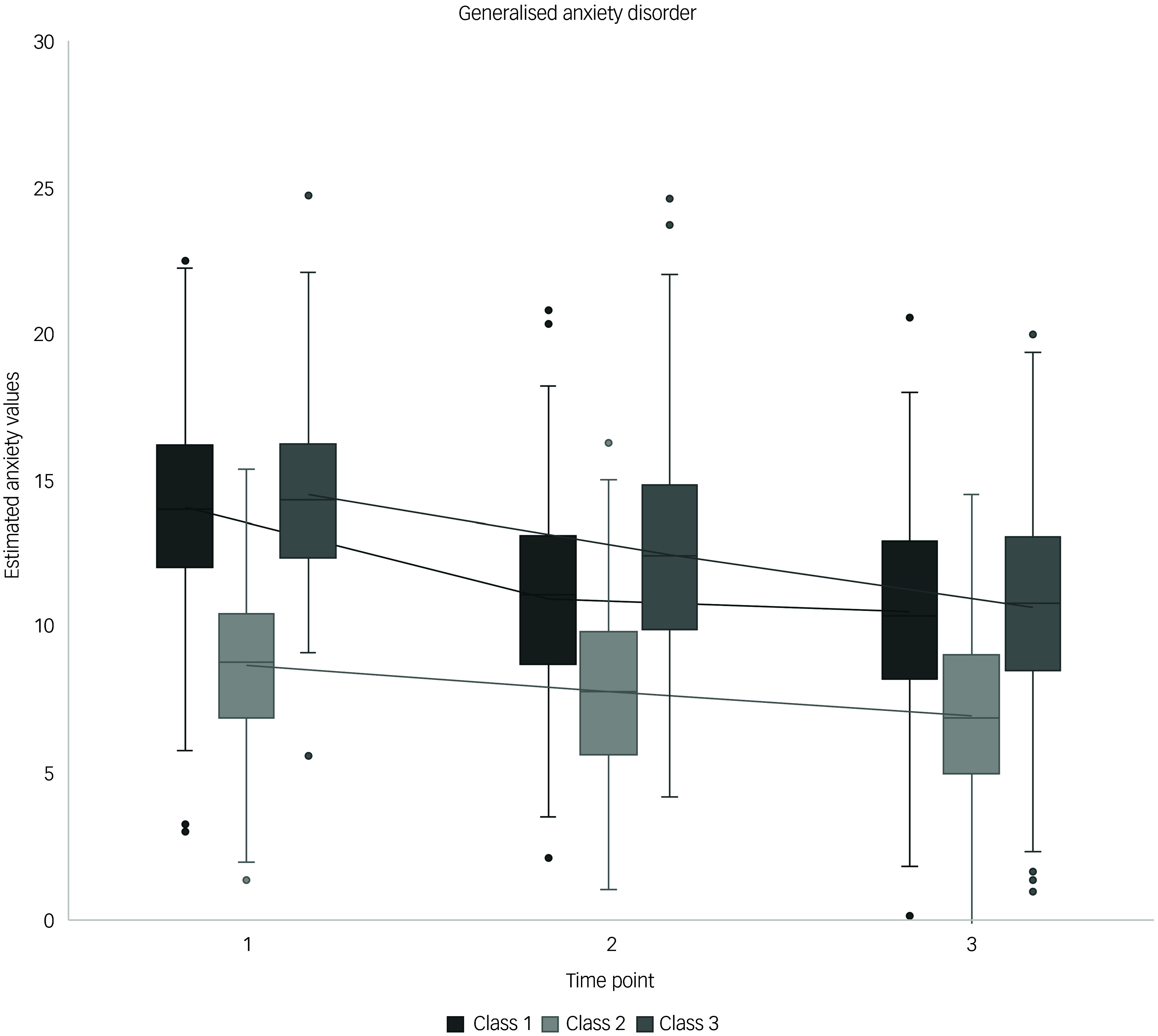
Estimated generalised anxiety disorder trajectories against time, controlled for self-efficacy and repetitive negative thinking. Class 1, low symptoms; class 2, recovery; class 3, distress. Time point 1, 4 weeks post-earthquake; time point 2, 11 weeks post-earthquake; time point 3: 18 weeks post-earthquake.

The analysis of GAD led to the identification of three trajectories, explaining 69.2% of the total variance (Supplementary Table 6): low symptom (class 1), recovery (class 2) and distress (class 3), with class distributions of 50.2, 28.2 and 21.6%, respectively. Participants in class 1 started with the lowest intercept (−1.77), followed by class 2 (3.75) and then class 3 (16.41). Time significantly differentiated class 1 from class 2. In class 1, symptoms decreased from T2 to T3, as indicated by negative coefficients, whereas similar decreases were observed in classes 2 and 3 only at T3. Self-efficacy emerged as a class-differentiating factor, with a positive coefficient in class 1 but negative coefficients in classes 2 and 3. RNT was a significant predictor across all classes, showing positive coefficients, although it did not significantly differentiate between classes. Trauma history significantly influenced class membership, with elevated trauma exposure increasing the likelihood of belonging to class 1 and decreasing that of being in classes 2 and 3. More years of education slightly increased the likelihood of belonging to classes 2 and 3 but reduced that of belonging to class 1. Figure 3 illustrates the estimated GAD trajectories over time, adjusted for self-efficacy and RNT.

## Discussion

The Kahramanmaraş earthquakes hit a population already affected by ongoing armed conflict. This is the first longitudinal investigation initiated within 4 weeks of a natural disaster to examine its mental health effects in a full-scale conflict-affected region. A significant proportion of participants were living in tents, with most reporting high exposure to potentially traumatic events. Our findings reveal high prevalences of PTSD, depression and GAD, along with high comorbidity rates, with more than 50% of participants exceeding probable diagnostic criteria according to self-report cut-offs at all three time points. These prevalences surpass those reported by previous studies in regions affected by both natural disasters and armed conflicts, such as Vietnam^2,3^ and Pakistan,^4^ and are higher than those reported in Turkey or west Syria^12^ 40 days post-earthquake. They are also higher than pooled prevalences across different countries with a recent war history.^34^ The elevated prevalences may be attributed to the proximity of time to the event and the ongoing sociopolitical challenges the population has endured, which have also hindered immediate relief efforts. This highlights the urgent need for timely, tailored, evidence-based interventions to address the specific mental health needs of survivors and mitigate long-term psychopathology.

To discern mental health needs more precisely, we conducted LCGA up to 18 weeks post-earthquakes. In line with previous longitudinal studies conducted in similar settings,^8,9^ we found that a substantial percentage of participants followed a pattern of psychological resilience, with low-symptom and recovery trajectories for PTSD, depression and GAD. Nonetheless, the prevalences of mental disorders remained concerningly high over the course of 18 weeks. Notably, RNT exacerbated symptom severity; it is likely to maintain psychological distress in war-affected populations by reinforcing attentional biases toward negative events and maladaptive cognitive processing, thereby leading to heightened emotional reactivity and compromised coping mechanisms.^15,17^ However, individuals with greater trauma exposure were more likely to follow a recovery trajectory for PTSD and depression and a low-symptom trajectory for GAD, contradicting previous findings linking trauma exposure to greater distress.^8,9^ In addition, gender did not significantly influence psychopathology; this represented further divergence from previous findings.^8^ These unexpected outcomes highlight the need for more nuanced research to better understand risk factors in affected survivors.

The low-symptom PTSD trajectory, characterised by the lowest initial symptom level, was associated with lesser trauma exposure compared with the recovery trajectory. This result corroborates previous findings in regions affected by both conflict and natural disasters in LMICs and highlights the association between trauma exposure and psychopathology.^8,9^ However, education was not associated with trajectory membership in PTSD, unlike in depression and GAD. Further, the high-distress trajectory was not clearly predicted by RNT, nor was it linked to trauma history. These findings suggest alternative mechanisms that require further investigation.

For depression, all trajectories started with similar symptom severity, but RNT had a differential impact, worsening symptoms to varying degrees. The predominant trauma-sensitive recovery trajectory mirrored the recovery trajectory observed in PTSD, being characterised by recovery despite high trauma exposure. By contrast, participants showing the RNT-sensitive recovery trajectory had the lowest trauma exposure, the lowest education level and the greatest impact of RNT, suggesting that individuals with a combination of low trauma exposure and low education levels are more vulnerable to RNT.

In GAD, the predominant low-symptom trajectory was associated with the greatest trauma exposure and lowest education levels. Notably, self-efficacy was linked to increased symptoms in this trajectory, potentially indicating that high levels of self-efficacy were challenged by the uncontrollable realities of disaster aftermath.^35^ This finding was in contrast to the protective role of self-efficacy observed in the recovery and distress trajectories.^14^ Similar to the findings for depression, lower education levels in the low-symptom trajectory were associated with greater vulnerability to RNT, further suggesting that lower education levels might increase vulnerability to RNT across different psychopathologies. Our findings on depression and GAD align with the trajectories observed in a 12-month longitudinal study on anxiety and depression in Sri Lanka,^8^ in which low-symptom and recovery patterns were also identified. However, the majority of participants in our study followed recovery and low-symptom trajectories, despite the high prevalence of these conditions during the assessed period.

Our study has potential clinical implications. The high prevalence rates of PTSD, depression and GAD among war-affected populations also facing natural disasters indicates an urgent need for targeted research and interventions. The World Health Organization has developed scalable psychosocial interventions, such as Problem Management Plus and Step-by-Step, which have demonstrated effectiveness in distressed populations, particularly in LMICs.^36^ Our findings further emphasise the importance of addressing RNT as a significant factor in prolonged recovery across various mental disorders. The observed association of higher vulnerability to RNT with lower education levels, particularly among individuals with depression and GAD, requires additional examination. Furthermore, the complex relationship between self-efficacy and GAD trajectories also warrants further investigation to enhance the effectiveness of interventions. Although high levels of exposure to potentially traumatic events were anticipated in this population, the recovery and low symptom trajectories were associated with the highest levels of trauma exposure. This raises questions about the nature of the psychological resilience underlying these predominant trajectories. It is possible that our findings based on data collected within 18 weeks post-earthquake reflect an initial shock response, potentially overlooking any delayed development of psychopathology. In addition, improved housing conditions at T3 relative to T1, reported by 27.3% of participants, may have influenced these trajectories, although housing was not included as a covariate in our analysis. To address these questions, longer-term and more comprehensive assessments are needed to better understand the factors contributing to resilience and recovery in this context.^36^

Our study had several limitations that mean caution is required when interpreting the findings. We initially intended to use CLPA but switched to LCGA owing to inadequate model fit, without conducting power analyses for either method. The use of non-probability sampling and the exclusion of participants raise data quality concerns, further limiting the robustness of the results. In addition, the validity of self-reported assessments of mental disorders, RNT and self-efficacy may be compromised by factors such as bias and ambiguous questions. Self-efficacy and RNT were likely to have been influenced by experiences related to prolonged conflict; accordingly, our measurements probably represent dispositional characteristics that were formed before the disaster. Further, the complex social ecology in northwest Syria following the earthquakes posed distinct challenges that may have exacerbated mental health vulnerability beyond the effects of individual cognitive factors. Delayed aid, political instability and conflict-ridden conditions caused prolonged displacement, loss of shelter and restricted healthcare access, probably increasing the prevalence of mental disorders.^37,38^ Future research should consider socioecological factors such as housing conditions to more accurately assess the predictive role of cognitive factors in mental health outcomes. Finally, there may be potential selection bias, as individuals who have repeatedly experienced harsh conditions may be healthier and more likely to recover from mental health problems.

Our findings shed light on the mental health status of high-adversity populations that are further affected by natural disasters; they demonstrate high prevalences of PTSD, depression and GAD in survivors of war and natural disasters and also provide novel insights into the different trajectories of psychopathology in this population. Cumulative traumas combined with RNT were associated with elevated initial distress and protracted recovery trajectories. Future research should further evaluate the role of cognitive factors in individuals faced with multiple types of adversity.

## Supporting information

Churbaji et al. supplementary material Churbaji et al. supplementary material

## Data Availability

Deidentified participant data, analytic code and research material in anonymised form will be available after publication from the corresponding author (D.C.) on reasonable request.
