# Supplementary material for: When an earthquake hits a war-affected population: longitudinal growth trajectories of psychopathology in individuals in northwest Syria
Source: Br J Psychiatry. 2025 Apr 21;228(3):220–8. doi: 10.1192/bjp.2025.1 (PMC12912877; doi:10.1192/bjp.2025.1)
Supplement: Churbaji et al. supplementary material [file S0007125025000017sup001.pdf]

Supplementary material

When an earthquake hits a war-affected population. Longitudinal growth trajectories of psychopathology in individuals in Northwest Syria

Dana Churbaji[1], PhD, Linnea Ritter[1], MSc, Pascal Schlechter[1], PhD, Ahlke Kip[1], PhD, & Nexhmedin Morina[1,2], PhD

[1] Institute of Psychology, University of Münster, Münster Germany

[2] Department of Psychology, New School for Social Research, New York, USA

*Corresponding Author, Dr. Dana Churbaji, Institute of Psychology, University of Münster, Fliednerstr.21, 48149 Münster, Germany, Tel: +492518339124, Fax: +492518331331, e-Mail: churbaji@uni-muenster.de

Supplementary materials: Supplementary Tables S1-S6

SUPPLEMENTARY TABLE S1: INTERNAL CONSISTENCIES (CRONBACH'S ALPHA) OF THE INCLUDED SCALES 3

SUPPLEMENTARY TABLE S2: PEARSON CORRELATION COEFFICIENTS OF KEY VARIABLES 4

SUPPLEMENTARY TABLE S3: COMPARISON OF FIT INDICES FOR LATENT CLASS GROWTH MODELS WITH 1-5 CLASSES FOR PTSD, DEPRESSION, AND GENERALIZED ANXIETY AT THREE TIME POINTS. 4

SUPPLEMENTARY TABLE S4: POST-TRAUMATIC STRESS DISORDER PARAMETER ESTIMATES PER CLASS AND PAIRED COMPARISONS ASSESSED BY THE INTERNATIONAL TRAUMA QUESTIONNAIRE (ITQ). 5

SUPPLEMENTARY TABLE S5: DEPRESSION PARAMETER ESTIMATES PER CLASS AND PAIRED COMPARISONS ASSESSED BY THE PATIENT HEALTH QUESTIONNAIRE-8 (PHQ-8) 7

SUPPLEMENTARY TABLE S6: GENERALIZED ANXIETY DISORDER PARAMETER ESTIMATES PER CLASS AND PAIRED COMPARISONS ASSESSED BY THE GENERALIZED ANXIETY DISORDER SCALE-7 (GAD-7) 7

Supplementary Table S1

Internal consistencies (Cronbach's alpha) of the included scales.

	T1	T2	T3
ITQ	0.81	0.85	0.85
PHQ-8	0.83	0.88	0.83
GAD7	0.88	0.91	0.91
GSE	0.90	0.94	0.95
PTQ	0.85	0.87	0.85

Note. ITQ=International Trauma Questionnaire. PHQ-8=Patient Health Questionnaire-8. GAD-7=Generalized Anxiety Disorder scale-7. GSE=General Self-Efficacy scale. PTQ=Perseverative Thinking Questionnaire.

Supplementary Table S2

Pearson Correlation Coefficients of Key Variables.

	ITQ T1	ITQ T2	ITQ T3	PHQ-8 T1	PHQ-8 T2	PHQ-8 T3	GAD-7 T1	GAD-7 T2	GAD-7 T3	GSE T1	GSE T2	GSE T3	PTQ T1	PTQ T2	PTQ T3	RTHC	Gender
ITQ T2	.456**	–	–	–	–	–	–	–	–	–	–	–	–	–	–	–	–
ITQ T3	.446**	.708**	–	–	–	–	–	–	–	–	–	–	–	–	–	–	–
PHQ-8 T1	.491**	.380**	.322**	–	–	–	–	–	–	–	–	–	–	–	–	–	–
PHQ-8 T2	.331**	.648**	.559**	.374**	–	–	–	–	–	–	–	–	–	–	–	–	–
PHQ-8 T3	.219**	.470**	.484**	.346**	.499**	–	–	–	–	–	–	–	–	–	–	–	–
GAD-7 T1	.510**	.395**	.375**	.712**	.398**	.367**	–	–	–	–	–	–	–	–	–	–	–
GAD-7 T2	.325**	.586**	.568**	.383**	.791**	.524**	.456**	–	–	–	–	–	–	–	–	–	–
GAD-7 T3	.333**	.467**	.563**	.357**	.486**	.803**	.420**	.510**	–	–	–	–	–	–	–	–	–
GSE T1	.137	.005	.031	.068	.004	-.031	.129	.063	-.017	–	–	–	–	–	–	–	–
GSE T2	-.032	.074	.101	-.146*	.040	-.094	-.121	-.022	-.139	.275**	–	–	–	–	–	–	–
GSE T3	-.180*	-.003	-.056	-.158*	-.081	-.153*	-.098	-.161*	-.227**	.279**	.336**	–	–	–	–	–	–
PTQ T1	.554**	.335**	.352**	.537**	.353**	.312**	.549**	.381**	.352**	.171*	-.183**	-.111	–	–	–	–	–
PTQ T2	.236**	.604**	.560**	.289**	.731**	.581**	.317**	.634**	.526**	-.002	-.024	-.110	.422**	–	–	–	–
PTQ T3	.203**	.498**	.555**	.280**	.565**	.728**	.295**	.572**	.724**	-.049	-.103	-.164*	.381**	.758**	–	–	–
RTHC	.144*	.134	.106	.134	.165*	.258**	.151*	.167*	.200**	-.120	-.056	-.168*	.039	.117	.146	–	–
Gender	.006	-.015	-.012	.025	-.015	-.001	.055	-.057	.082	-.067	-.053	.032	.125	.061	.176*	-.097	–
Education	-.146*	.025	-.047	.018	.038	.020	-.059	.025	-.007	.080	.000	.048	-.042	.017	.122	.128	.112

Note. ITQ=International Trauma Questionnaire. PHQ-8=Patient Health Questionnaire-8. GAD-7=Generalized Anxiety Disorder scale-7. GSE=General Self-Efficacy scale. PTQ=Perseverative Thinking Questionnaire. RTHC=Refugee Trauma History Checklist.

* *p* < .05, ** *p* < .01, *** *p* < .001.

Supplementary Table S3

Comparison of fit indices for latent class growth models with 1-5 classes for PTSD, depression, and generalized anxiety at three time points.

Number of classes	BIC	AIC	AIC3	Npar	VLMR	*p*-value	Class.Err.	Entropy R^2	R^2
PTSD (ITQ)									
1	3216.03	3196.12	3202.12	6	–	–	0.00	1.00	0.39
2	3183.21	3130.12	3146.12	16	86.00	0.00	0.12	0.60	0.66
**3**	**3194.01**	**3107.74**	**3133.74**	**26**	**42.38**	**<0.001**	**0.11**	**0.68**	**0.68**
4	3221.39	3101.94	3137.94	36	25.80	0.12	0.19	0.62	0.71
5	3242.84	3090.21	3136.21	46	31.74	0.11	0.12	0.72	0.72
Depression (PHQ-8)									
1	3268.84	3248.93	3254.93	6	–	–	0.00	1.00	0.50
2	3263.89	3210.80	3226.80	16	58.13	<0.001	0.09	0.63	0.65
**3**	**3280.42**	**3194.15**	**3220.15**	**26**	**36.66**	**<0.01**	**0.09**	**0.75**	**0.65**
4	3310.72	3191.27	3227.27	36	22.88	0.14	0.14	0.65	0.71
5	3332.47	3179.84	3225.84	46	31.43	0.03	0.14	0.73	0.72
Generalized Anxiety (GAD-7)									
1	3251.55	3231.64	3237.64	6	–	–	0.00	1.00	0.44
2	3239.74	3186.65	3202.65	16	64.99	<0.001	0.10	0.62	0.64
**3**	**3247.96**	**3161.69**	**3187.69**	**26**	**44.96**	**<0.001**	**0.16**	**0.63**	**0.69**
4	3254.42	3134.97	3170.97	36	46.72	<0.001	0.22	0.61	0.73
5	3274.53	3121.89	3167.89	46	33.07	<0.001	0.17	0.71	0.75

Note. Bold style indicates best-fitting model. ITQ=International Trauma Questionnaire. PHQ-8=Patient Health Questionnaire-8. GAD-7=Generalized Anxiety Disorder scale-7.

Supplementary Table S4

Post-traumatic stress disorder parameter estimates per class and paired comparisons assessed by the International Trauma Questionnaire (ITQ).

	Parameters					Paired comparisons: Wald (df)		
	Class 1 n=122.2	Class 2 n=72.5	Class 3 n=9.0	Wald	Wald(=)	Classes 1,2	Classes 1,3	Classes 2,3
Model for dependent								
R^2	0.50	0.41	0.03	–	–	–	–	–
Intercept	5.59	0.95	16.77	71.78***	27.46***	5.60 (1)*	16.60 (1) ***	27.46 (1) ***
Time	–	–	–	71.14***	8.18	0.04 (2)	7.64* (2)	6.24* (2)
1	1.58	1.52	-0.12	–	–	–	–	–
2	-0.68	-0.71	0.22	–	–	–	–	–
3	-0.90	-0.81	-0.1	–	–	–	–	–
GSE	0.05	0.11	0.10	7.46	0.8	0.74 (1)	0.13(1)	0.00 (1)
RNT	0.21	0.18	-0.03	239.73***	13.44**	1.22 (1)	13.12 (1)***	9.18 (1)**
Model for classes								
Intercept	-0.36	-0.35	0.71	0.22	–	0.00 (1)	0.2 (1)	0.21 (1)
Covariates								
RTHC	0.41	0.09	-0.50	19.13***	–	12.53 (1)***	12.11 (1)***	5.55 (1)*
Gender	–	–	–	2.37	–	0.17 (1)	2.34 (1)	2.16 (1)
M	-0.7	-0.61	1.31	–	–	–	–	–
F	0.7	0.61	-1.31	–	–	–	–	–
Education	-0.03	0.11	-0.09	3.55	–	2.83 (1)	0.11 (1)	1.25 (1)

Note. GSE=General Self-Efficacy scale, RNT=Repetitive Negative Thinking, RTHC=Refugee Trauma History Checklist. *p*-values are indicated as follows: *** p <.001, ** p <.01, * p <.05.

Supplementary Table S5

Depression parameter estimates per class and paired comparisons assessed by the Patient Health Questionnaire-8 (PHQ-8).

	Parameters					Paired comparisons: Wald (df)		
	Class 1 n=129.3	Class 2 n=41.9	Class 3 n=32.8	Wald	Wald(=)	Classes 1,2	Classes 1,3	Classes 2,3
Model for dependent								
R^2	0.48	0.79	0.45	–	–	–	–	–
Intercept	3.07	2.14	3.87	11.01*	0.28	0.15 (1)	0.08 (1)	0.26 (1)
Time	–	–	–	57.59***	2.33	0.12 (2)	1.99 (2)	1.66 (2)
1	1.68	1.63	0.82	–	–	–	–	–
2	-0.82	-0.95	-0.35	–	–	–	–	–
3	-0.87	-0.68	-0.48	–	–	–	–	–
GSE	0.03	-0.12	-0.08	5.01	4.24	3.36 (1)	1.60 (1)	0.16 (1)
RNT	0.27	0.4	0.18	525.67***	32.57***	18.79 (1)***	5.69 (1)*	28.57 (1)***
Model for classes								
Intercept	-5.93	7.24	-1.30	10.36**	–	10.36 (1)**	3.73 (1)	6.80 (1)**
Covariates								
RTHC	0.85	-0.95	0.09	14.81***	–	13.44(1)***	8.50 (1)**	6.16 (1)*
Gender	–	–	–	2.85	–	0.40(1)	0.98 (1)	2.26 (1)
M	0.004	0.36	-0.36	–	–	–	–	–
F	-0.004	-0.36	0.36	–	–	–	–	–
Education	0.23	-0.32	0.09	5.63	–	5.57 (1)*	1.08 (1)	4.37 (1)*

Note. GSE=General Self-Efficacy scale, RNT=Repetitive Negative Thinking, RTHC=Refugee Trauma History Checklist. *p*-values are indicated as follows: *** p <.001, ** p <.01, * p <.05.

Supplementary Table S6

Generalized anxiety disorder parameter estimates per class and paired comparisons assessed by the Generalized Anxiety Disorder scale-7 (GAD-7).

	Parameters					Paired comparisons: Wald (df)		
	Class 1n=102.6	Class 2n=57.3	Class 3 n=44.1	Wald	Wald(=)	Classes 1,2	Classes 1,3	Classes 2,3
Model for dependent								
R^2	0.55	0.55	0.59	–	–	–	–	–
Intercept	-1.77	3.75	16.41	74.61***	62.92***	3.86 (1)*	62.09 (1)***	18.64 (1)***
Time	–	–	–	45.80***	8.91	6.64 (2)*	1.59 (2)	4.41 (2)
1	1.58	0.31	1.43	–	–	–	–	–
2	-0.67	0.14	0.04	–	–	–	–	–
3	-0.91	-0.45	-1.47	–	–	–	–	–
GSE	0.17	-0.14	-0.41	48.11***	47.45***	10.48 (1)***	46.58 (1)***	7.13 (1)**
RNT	0.27	0.23	0.22	371.24***	2.44	1.37 (1)	2.18 (1)	0.19 (1)
Model for classes								
Intercept	-0.04	0.17	-0.13	0.06	–	0.02 (1)	0.00 (1)	0.04 (1)
Covariates								
RTHC	0.43	-0.15	-0.27	15.98***	–	15.47 (1)***	10.90 (1)***	0.63 (1)
Gender	–	–	–	3.90	–	1.26 (1)	3.69 (1)	2.30 (1)
M	-0.43	-0.02	0.44	–	–	–	–	–
F	0.43	0.02	-0.44	–	–	–	–	–
Education	-0.22	0.08	0.14	5.98*	–	5.07 (1)*	4.69 (1)*	0.18 (1)

Note. GSE=General Self-Efficacy scale, RNT=Repetitive Negative Thinking, RTHC=Refugee Trauma History Checklist.

p-values are indicated as follows: *** $p < .001$, ** $p < .01$, * $p < .05$
